# Supplementary material for: Spontaneous base flipping helps drive Nsp15’s preferences in double stranded RNA substrates
Source: Nat Commun. 2025 Jan 4;16:391. doi: 10.1038/s41467-024-55682-0 (PMC11700208; doi:10.1038/s41467-024-55682-0)
Supplement: Supplementary file 1 — Supplementary Information [file 41467_2024_55682_MOESM1_ESM.pdf]

Supplementary Materials

Spontaneous base flipping helps drive Nsp15′s preferences in double stranded RNA substrates

Zoe M. Wright[1], Kevin John Butay[2,3], Juno M. Krahn[2], Isha M. Wilson[1,4], Scott A. Gabel,[2] Eugene F. DeRose,[2] Israa S. Hissein[1], Jason G. Williams[5], Mario J. Borgnia[2], Meredith N. Frazier[1,6], Geoffrey A. Mueller[2], and Robin E. Stanley[1*]

[1]Signal Transduction Laboratory, National Institute of Environmental Health Sciences, National Institutes of Health, Department of Health and Human Services, 111 T. W. Alexander Drive, Research Triangle Park, NC 27709, USA

[2]Genome Integrity and Structural Biology Laboratory, National Institute of Environmental Health Sciences, National Institutes of Health, Department of Health and Human Services, 111 T. W. Alexander Drive, Research Triangle Park, NC 27709, USA

[3]Current Affiliation: Department of Biochemistry, Duke University, Durham, NC 27710, USA

[4]Current Affiliation: Howard University College of Medicine, Washington, DC 20059, USA

[5]Epigenetics and Stem Cell Biology Laboratory, National Institute of Environmental Health Sciences, National Institutes of Health, Department of Health and Human Services, 111 T. W. Alexander Drive, Research Triangle Park, NC 27709, USA

[6]Department of Chemistry and Biochemistry, College of Charleston, 66 George St., Charleston, SC, USA 29424

*Correspondence and requests for materials should be addressed to (email: zoe.wright@nih.gov; robin.stanley@nih.gov)

This File Includes:

Supplementary Tables 1–5

Supplementary Figures 1–10

Supplementary Table 1: List of RNA oligos used for nuclease assays and cryo-EM experiments.

Oligo Name	5′ label	Sequence (5′ to 3′)	3′ label	Length (nt)	Used in Figures
1t	Cy5	$\mathrm{GACAU^FU^FU^FU^FAGU^FU^FU^FGU^FU^FAA\mathbf{U}U^FU^FAGAU^FGAAAU^FCU^FAAA}$	Fl	35	1, 4, SA, S4, S5
1c_A		UUUAGAUUUCAUCUAA**A**UUAACAAACUAAAAUGUC		35	1, 4, S4, S5
1c_G		UUUAGAUUUCAUCUAA**G**UUAACAAACUAAAAUGUC		35	1, S4, S5
1c_C		UUUAGAUUUCAUCUAA**C**UUAACAAACUAAAAUGUC		35	1, S4, S5
1c_U’		$\mathrm{UUUAGAUUUCAUCUAA\mathbf{U}^FUUAACAAACUAAAAUGUC}$		35	1, SA, S4, S5
1c_unpaired		UUUAGAUUUCAUCUAAUUAACAAACUAAAAUGUC		34	1, 4, S4, S5
1c_U		UUUAGAUUUCAUCUAA**U**UUAACAAACUAAAAUGUC		35	1, SA, S4, S5
TRSt	Cy5	UUUAGAUUUCAUC**U**AAACGAACAAACUAAAAUGUC	Fl	35	2, 3
TRSc_A		GACAUUUUAGUUUGUUCGUUU**A**GAUGAAAUCUAAA		35	2
TRSc_C		GACAUUUUAGUUUGUUCGUUU**C**GAUGAAAUCUAAA		35	2
TRSc_unp		GACAUUUUAGUUUGUUCGUUUGAUGAAAUCUAAA		34	2,3
2t	Cy5	$\mathrm{GACAU^FU^FU^FU^FAGU^FU^FU^FGU^FU^FCG\mathbf{U}GCAGAU^FGAAAU^FCU^FAAA}$	Fl	35	4, S4, S5
2c_A		UUUAGAUUUCAUCUGC**A**CGAACAAACUAAAAUGUC		35	4, S4, S5
2c_unpaired		UUUAGAUUUCAUCUGCCGAACAAACUAAAAUGUC		34	4, S4, S5
3t	Cy5	$\mathrm{GACAU^FU^FU^FU^FAGU^FU^FU^FGU^FU^FAA\mathbf{U}GCAGAU^FGAAAU^FCU^FAAA}$	Fl	35	4, S4, S5
3c_A		UUUAGAUUUCAUCUGC**A**UUAACAAACUAAAAUGUC		35	4, S4, S5
3c_unpaired		UUUAGAUUUCAUCUGCUUAACAAACUAAAAUGUC		34	4, S4, S5
4t	Cy5	$\mathrm{GACAU^FU^FU^FU^FAGU^FU^FU^FGU^FU^FCG\mathbf{U}U^FU^FAGAU^FGAAAU^FCU^FAAA}$	Fl	35	4, S4, S5
4c_A		UUUAGAUUUCAUCUAA**A**CGAACAAACUAAAAUGUC		35	4, S4, S5
4c_G		UUUAGAUUUCAUCUAA**G**CGAACAAACUAAAAUGUC		35	S4, S5
4c_C		UUUAGAUUUCAUCUAA**C**CGAACAAACUAAAAUGUC		35	S4, S5
4c_U		$\mathrm{UUUAGAUUUCAUCUAA\mathbf{U}^FCGAACAAACUAAAAUGUC}$		35	S4, S5

4c_unpaired		UUUAGAUUUCAUCUAACGAACAAACUAAAAUGUC		34	4, S4, S5
5t	Cy5	GACAU^{F}U^{F}U^{F}U^{F}AGU^{F}U^{F}U^{F}GU^{F}U^{F}AA**U**AAAGAU^{F}GAAAU^{F}CU^{F}AAA	Fl	35	4, S4, S5
5c_A		UUUAGAUUUCAUCUUU**A**UUAACAAACUAAAAUGUC		35	4, S4, S5
5c_unpaired		UUUAGAUUUCAUCUUUUUAACAAACUAAAAUGUC		34	4, S4, S5
SL4	Cy5	CUGUGUGGCUGUCACUCGGCUGCAUGCUUAGUGCACUCACGCAGAAAA	Fl	48	5
SL4-1	Cy5	CUGUGUGGCUGUCACUCGGCAGCAAGCUUAGUGCACUCACGCAGAAAA	Fl	48	S6

2′-F-U are denoted as U^{F}.

2′-OH-U are denoted as U.

Target Us and their direct complements are bold and red.

“Target” strands contain “t” in the name, “complement” strands contain “c” in the name.

Supplementary Table 2. Shortest distances between key Nsp15 residues and dsRNA (contact surface areas greater than or equal to 10 as calculated by *dr_sasa*[2]).

RNA			Protein			Distance (Å)
Chain	Residue	Atom	Chain	Residue	Atom	
g	U1	O3′	e	T113	OG1	3.349
g	U1	C4′	e	L134	CD2	4.419
g	U1	O4′	e	N137	OD1	3.343
g	U2	OP1	e	K111	CD	3.266
g	U2	OP1	e	T113	OG1	3.195
g	A11	O4′	c	Q20	OE1	4.941
g	A11	O3′	c	Q19	OE1	5.149
g	C13	O2′	a	Y343	OH	3.828
g	C13	C1′	a	K345	NZ	2.972
g	C13	OP1	c	K13	NZ	4.197
g	U14	C6	a	Y343	CE2	4.096
g	U14	O4	a	K345	CG	3.389
g	A15	N1	a	W333	CZ3	3.506
g	A15	N3	a	E340	OE1	3.497
g	A15	OP1	a	T341	OG1	3.054
g	A15	OP1	a	Y343	CD1	3.212
g	A16	C1′	a	E340	OE2	3.797
g	A24	O2′	c	G147	CA	3.428
g	A24	O3′	c	S148	OG	4.609
g	A24	OP1	c	K150	NZ	5.034
g	A25	OP1	c	S148	OG	3.468
h	U-15	C5′	c	K65	NZ	4.698
h	U-21	O2	a	W333	CH2	3.218
h	G-22	N3	a	V318	CG2	4.788
h	G-22	N2	a	M331	CE	3.266
h	A-23	O2′	a	V318	CG1	5.059
h	A-23	N3	a	K345	NZ	5.342

RNA: chain g = target strand; chain h = complementary strand

Protein: chain a = protomer P1; chain c = protomer P2; chain e = protomer P4

Supplementary Table 3: List of RNA oligos used for ^{19}F NMR experiments.

Oligo Name	Sequence (5′ to 3′)	Length (nt)	Used in Figures…
1t	GACAUUUUAGUUUGUUAA**U^F**UUAGAUGAAAUCUAAA	35	4, S4, S5
1c_A	UUUAGAUUUCAUCUAA**A**UUAACAAACUAAAAUGUC	35	4, S4, S5
1c_G	UUUAGAUUUCAUCUAA**G**UUAACAAACUAAAAUGUC	35	4, S4, S5
1c_C	UUUAGAUUUCAUCUAA**C**UUAACAAACUAAAAUGUC	35	4, S4, S5
1c_U	UUUAGAUUUCAUCUAA**U**UUAACAAACUAAAAUGUC	35	4, S4, S5
1c_unpaired	UUUAGAUUUCAUCUAAUUAACAAACUAAAAUGUC	34	4, S4, S5
2t	GACAUUUUAGUUUGUUCG**U^F**GCAGAUGAAAUCUAAA	35	4, S4, S5
2c_A	UUUAGAUUUCAUCUGC**A**CGAACAAACUAAAAUGUC	35	4, S4, S5
2c_G	UUUAGAUUUCAUCUGC**G**CGAACAAACUAAAAUGUC	35	S4, S5
2c_C	UUUAGAUUUCAUCUGC**C**CGAACAAACUAAAAUGUC	35	S4, S5
2c_U	UUUAGAUUUCAUCUGC**U**CGAACAAACUAAAAUGUC	35	S4, S5
2c_unpaired	UUUAGAUUUCAUCUGCCGAACAAACUAAAAUGUC	34	4, S4, S5
3t	GACAUUUUAGUUUGUUAA**U^F**GCAGAUGAAAUCUAAA	35	4, S4, S5
3c_A	UUUAGAUUUCAUCUGC**A**UUAACAAACUAAAAUGUC	35	4, S4, S5
3c_G	UUUAGAUUUCAUCUGC**G**UUAACAAACUAAAAUGUC	35	S4, S5
3c_C	UUUAGAUUUCAUCUGC**C**UUAACAAACUAAAAUGUC	35	S4, S5
3c_U	UUUAGAUUUCAUCUGC**U**UUAACAAACUAAAAUGUC	35	S4, S5
3c_unpaired	UUUAGAUUUCAUCUGCUUAACAAACUAAAAUGUC	34	4, S4, S5
4t	GACAUUUUAGUUUGUUCG**U^F**UUAGAUGAAAUCUAAA	35	4, S4, S5
4c_A	UUUAGAUUUCAUCUAA**A**CGAACAAACUAAAAUGUC	35	4, S4, S5
4c_G	UUUAGAUUUCAUCUAA**G**CGAACAAACUAAAAUGUC	35	4, S4, S5
4c_C	UUUAGAUUUCAUCUAA**C**CGAACAAACUAAAAUGUC	35	4, S4, S5
4c_U	UUUAGAUUUCAUCUAA**U**CGAACAAACUAAAAUGUC	35	4, S4, S5
4c_unpaired	UUUAGAUUUCAUCUAACGAACAAACUAAAAUGUC	34	4, S4, S5
5t	GACAUUUUAGUUUGUUAA**U^F**AAAGAUGAAAUCUAAA	35	4, S4, S5
5c_A	UUUAGAUUUCAUCUUU**A**UUAACAAACUAAAAUGUC	35	4, S4, S5
5c_G	UUUAGAUUUCAUCUUU**G**UUAACAAACUAAAAUGUC	35	S4, S5

5c_C	UUUAGAUUUCAUCUUU**C**UUAACAAACUAAAAUGUC	35	S4, S5
5c_U	UUUAGAUUUCAUCUUU**U**UUAACAAACUAAAAUGUC	35	S4, S5
5c_unpaired	UUUAGAUUUCAUCUUUUUAACAAACUAAAAUGUC	34	4, S4, S5
SL4-U87	GGG**U^F**GUGGCUGUCACUCGGCUGCAUGCUUAGUGCACUCACGCCC	44	5
SL4-U95	GGGUGUGGCUG**U^F**CACUCGGCUGCAUGCUUAGUGCACUCACGCCC	44	5
SL4-U104	GGGUGUGGCUGUCACUCGGC**U^F**GCAUGCUUAGUGCACUCACGCCC	44	5
SL4-U112	GGGUGUGGCUGUCACUCGGCUGCAUGCU**U^F**AGUGCACUCACGCCC	44	5
SL4-U115	GGGUGUGGCUGUCACUCGGCUGCAUGCUUAG**U^F**GCACUCACGCCC	44	5

2′-F-U are denoted as U^F.

2′-OH-U are denoted as U.

Target Us and their direct complements are bold and red.

“Target” strands contain “t” in the name, “complement” strands contain “c” in the name.

Supplementary Table 4: ^{19}F NMR shifts and linewidths for all oligos.

Oligo		Shift (ppm, ^{19}F)	Linewidth (ppm, ^{19}F)
1	unpaired	-198.8	178
	U-A	-199.8	107
	U•U	-200	133
	U•C	-200.3	112
	U•G	-198.6	111
2	unpaired	-197.8	137
	U-A	-200.5	111
	U•U	-200.9	160
	U•C	-201	116
	U•G	-199.7	112
3	unpaired	-198.3	135
	U-A	-200.3	110
	U•U	-200.4	205
	U•C	-200.5	109
	U•G	-199.7	112
4	unpaired	-198.9	229
	U-A	-200.2	112
	U•U	-200.3	125
	U•C	-200.7	108
	U•G	-198.6	112
5	unpaired	-199.5	168
	U-A	-199.9	109
	U•U	-199.7	140
	U•C	-200.4	106
	U•G	-199.1	115
SL4-U87	U•G	-199.4	112
SL4-U95	unpaired (flipped)	-199.3	135
SL4-U104	unpaired (stacked)	-200.8	164
SL4-U112	U•C	-202.4	113
SL4-U115	U-A	-200.8	110

Supplementary Table 5: Fractional abundance of SL4 and SL4-1 cleavage products detected by mass spectrometry (MS).

Experiment	Residues	Measured Monoisotopic Mass	Predicted Monoisotopic Mass	Delta Mass (ppm)	Sum Intensity	Percent Abundance	# of Charge States	Charge State Distribution	Apex RT (min)
Intact SL4 (control, Figure S7)	Cy5-C84:A131-Fl	16492.341	16492.381	2.425	3.31E+03	**100.00**	6	14 – 20	13.16
SL4 Nsp15 digest (Figures 5, S7)	Cy5-C84:U95 3′ cyclic phosphate	4397.712	4397.691	4.772	7.79E+05	**87.56**	3	3 – 5	8.38
	Cy5-C84:U95 3′ phosphate	4415.724	4415.701	5.148	8.58E+04	**9.65**	2	3 – 4	7.83
	Cy5-C84:U95 3′ cyclic phosphate + Na	4419.710	4419.673	8.409	1.24E+04	**1.40**	2	3 – 4	8.37
	Cy5-C84:C100 3′ cyclic phosphate	5947.922	5947.891	5.225	5.92E+03	**0.67**	1	4 – 4	9.60
	HO-C121:A131-Fl	4125.712	4125.694	4.339	1.20E+03	**0.14**	1	4 – 4	8.55
	Cy5-C84:U104 3′ cyclic phosphate	7249.075	7249.051	3.380	7.71E+02	**0.09**	1	5 – 5	9.90
	HO-A113:A131-Fl	6696.032	6696.025	1.045	N/A†	**<0.05†**	1	5 – 5	~9.85†
SL4-1 Nsp15 digest (Figure S6)	Cy5-C84:U95 3′ cyclic phosphate	4397.713	4397.691	5.082	2.70E+05	**95.40**	3	3 – 5	8.37
	Cy5-C84:U95 3′ cyclic phosphate +Na	4419.694	4419.673	4.642	1.14E+03	**0.40**	2	3 – 4	8.35

This table shows the oligos found with BioPharma Finder (Thermo Fisher Scientific, Waltham, MA, USA) analyses of the MS data from intact SL4 sample as well as the Nsp15 treated SL4 and SL4-1 samples. While the C1:C12 fragment is by far the most readily detected fragment identified (>95% fractional abundance) in the Nsp15 treated samples, it should be noted that larger oligonucleotides are less readily detected in these analyses and smaller oligonucleotides may be somewhat over-represented.

See Source Data file for oligo sequences and a full list of theoretical masses.

† Fragment A113:A131 was detected manually but with insufficient abundance for BioPharma Finder to generate a fractional abundance. See Supplemental Figure S8 panel D for mass spectrum.

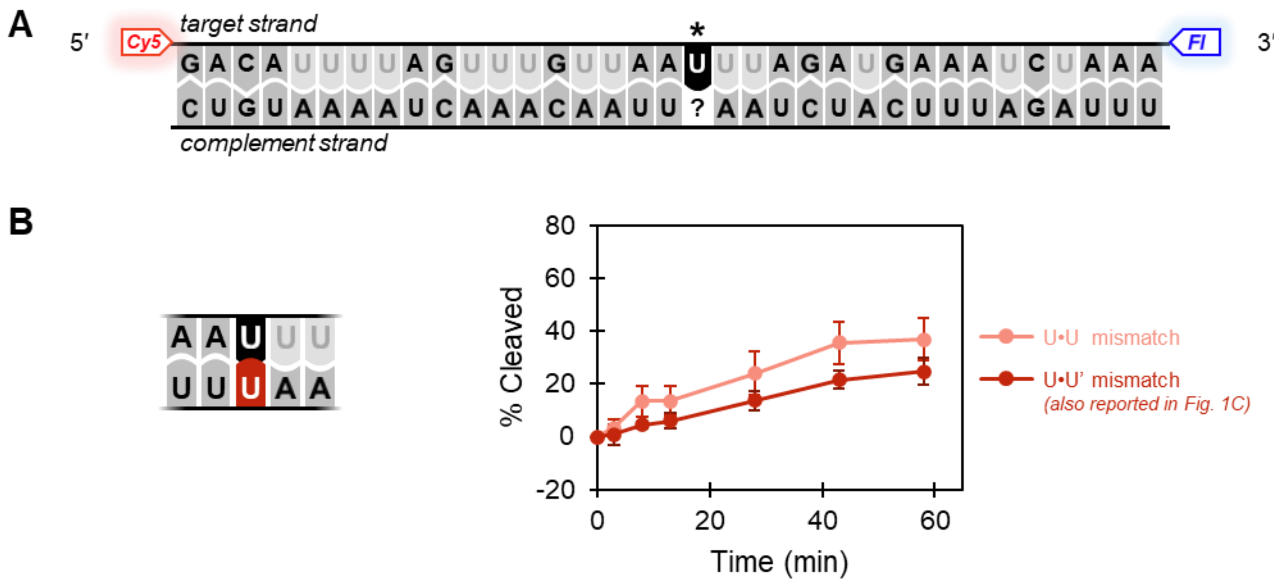

Supplementary Figure 1. A) dsRNA oligo substrate design. The target strand, containing U19 (marked with *) is labeled with 5′ Cy5 and 3′ Fl. Us in the target strand other than U19 have been substituted for 2′-F-U (light gray) which is not cleavable by Nsp15. The complement strand is unlabeled. B) Percent of target strand cleaved over time, quantified via the intensity of the uncleaved RNA band and normalized to the 2 min timepoint. Each point with error bars represents average and standard deviation for at least three independent reactions (N = 3 biological replicates). Images of each gel and quantification data are provided in the Source Data file. In the U•U mismatch substrate, both U19 in the target strand and its complement (red U) are cleavable; in the U•U’ mismatch substrate, which is also reported in Figure 1C and has been reproduced here to facilitate comparison, U19 in the target strand is cleavable but its complement is an uncleavable 2′-F-U.

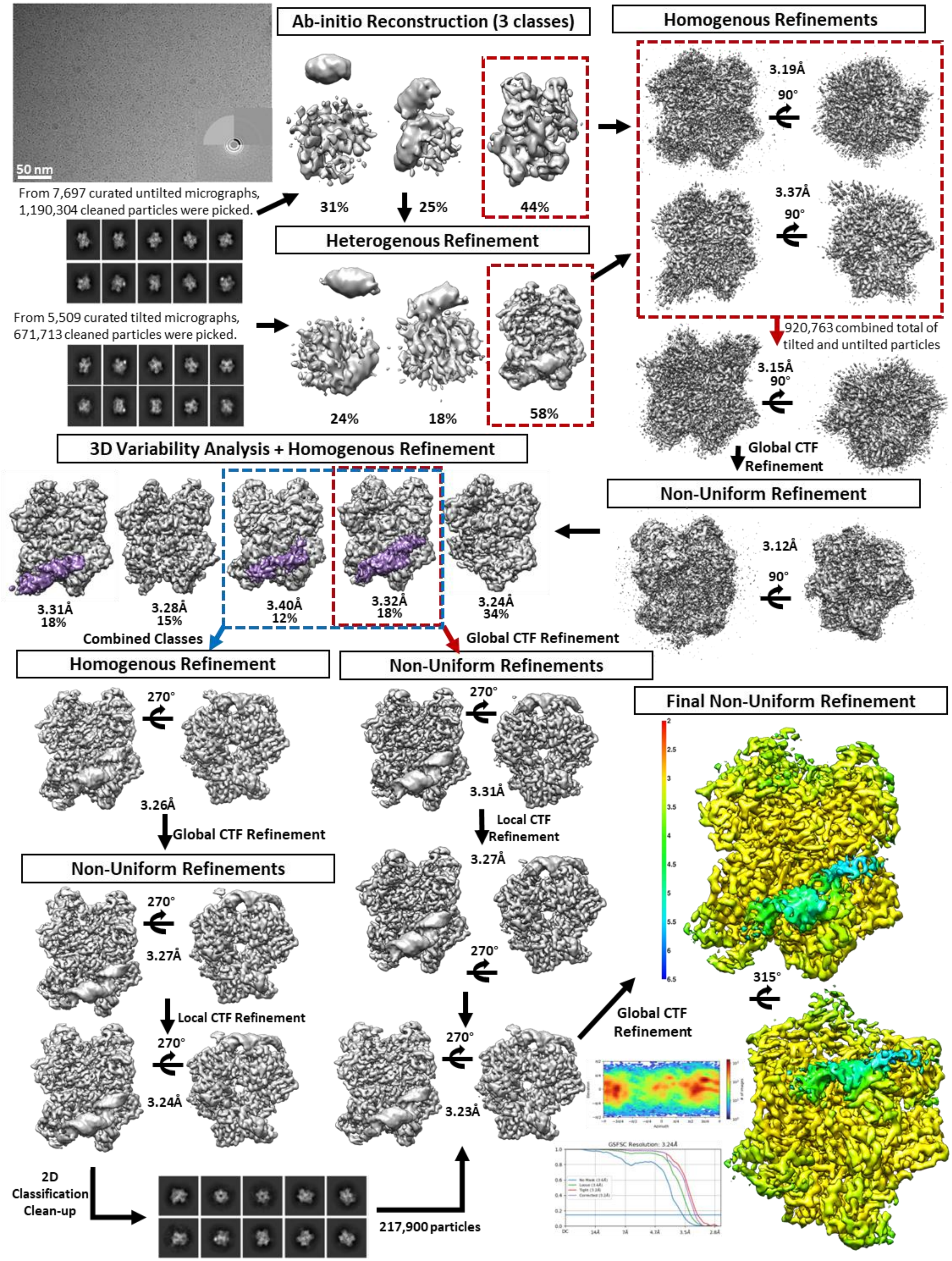

Supplementary Figure 2. Cryo-EM data processing workflow, performed in CryoSPARC.[1]

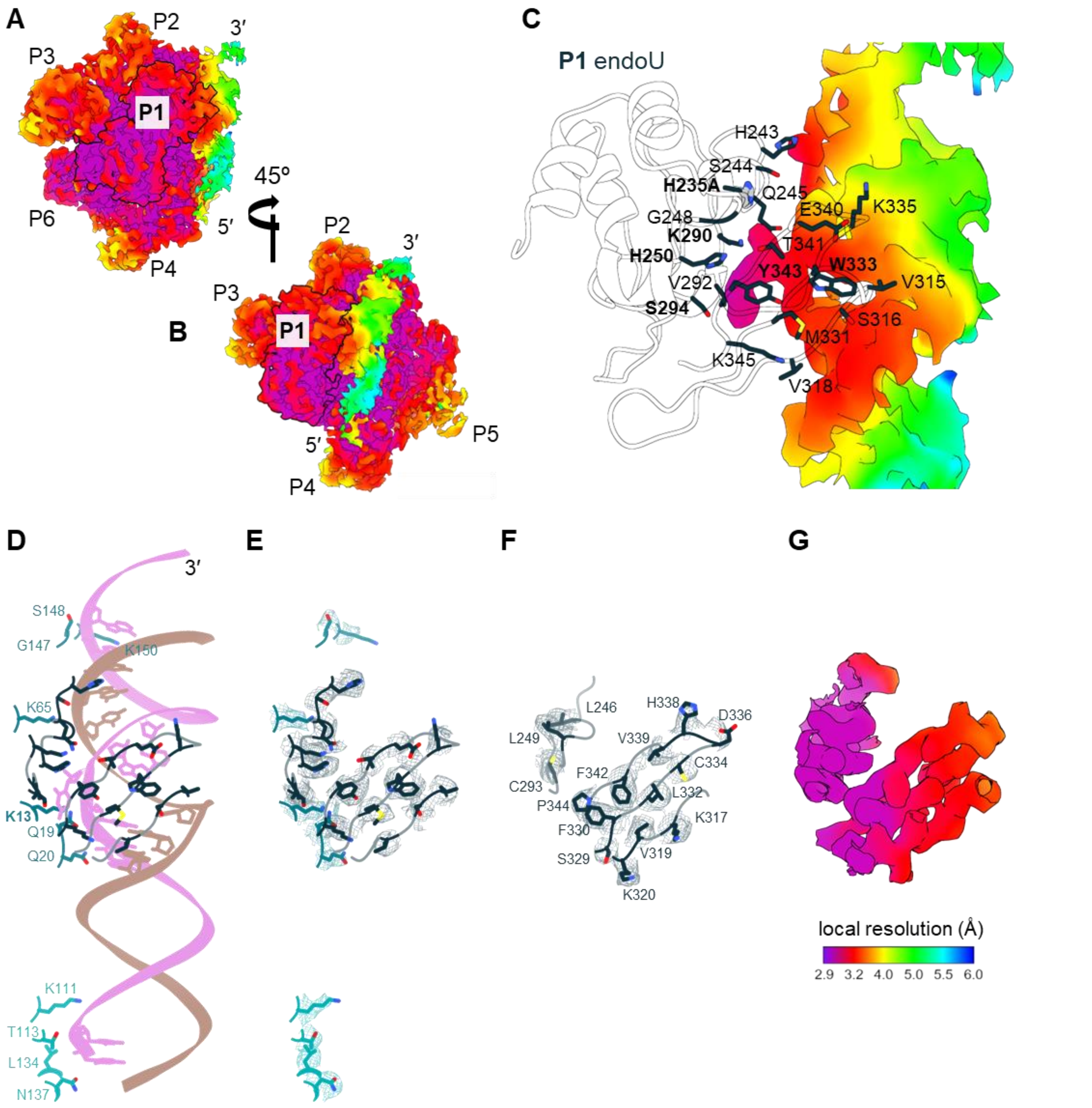

Supplementary Figure 3. Details of cryo-EM density map local resolution and atomic model of Nsp15-bound dsRNA. A) reproduced from main text Figure 3D to facilitate comparison with B), a rotated view (45° clockwise around y axis) showing protomer P5. C) Expanded version of Figure 3E showing all residues in P1 endoU calculated by dr_sasa[2] to interact with the dsRNA. D) Atomic model of dsRNA and residues of Nsp15 calculated by dr_sasa[2] to interact with dsRNA, highlighting residues from protomers P2 (top and center, in mid-blue) and P4 (bottom, in turquoise). Residues from protomer P1 are present (center, in black) but not labeled. K13, in the N-terminal domain of P2, encloses the flipped U of the dsRNA in the active site, helping to stabilize both the hydrophobic face of the flipped U and the charged phosphate backbone. All other residues shown for P2 and P4 interact with the dsRNA exclusively via the sugar-phosphate backbone. E) Interacting resides of Nsp15 (same as in D) with mesh cryo-EM density map for sidechains. F) Residues of protomer P1 located between those highlighted in panels C/D/E, with mesh cryo-EM density map for sidechains. These residues do not form direct contacts with the dsRNA, but do form a secondary shield of hydrophobic and positively charged residues. G) Cryo-EM density map of a portion of the endoU domain of P1 (corresponding to residues and view highlighted in panels E+F), colored by local resolution.

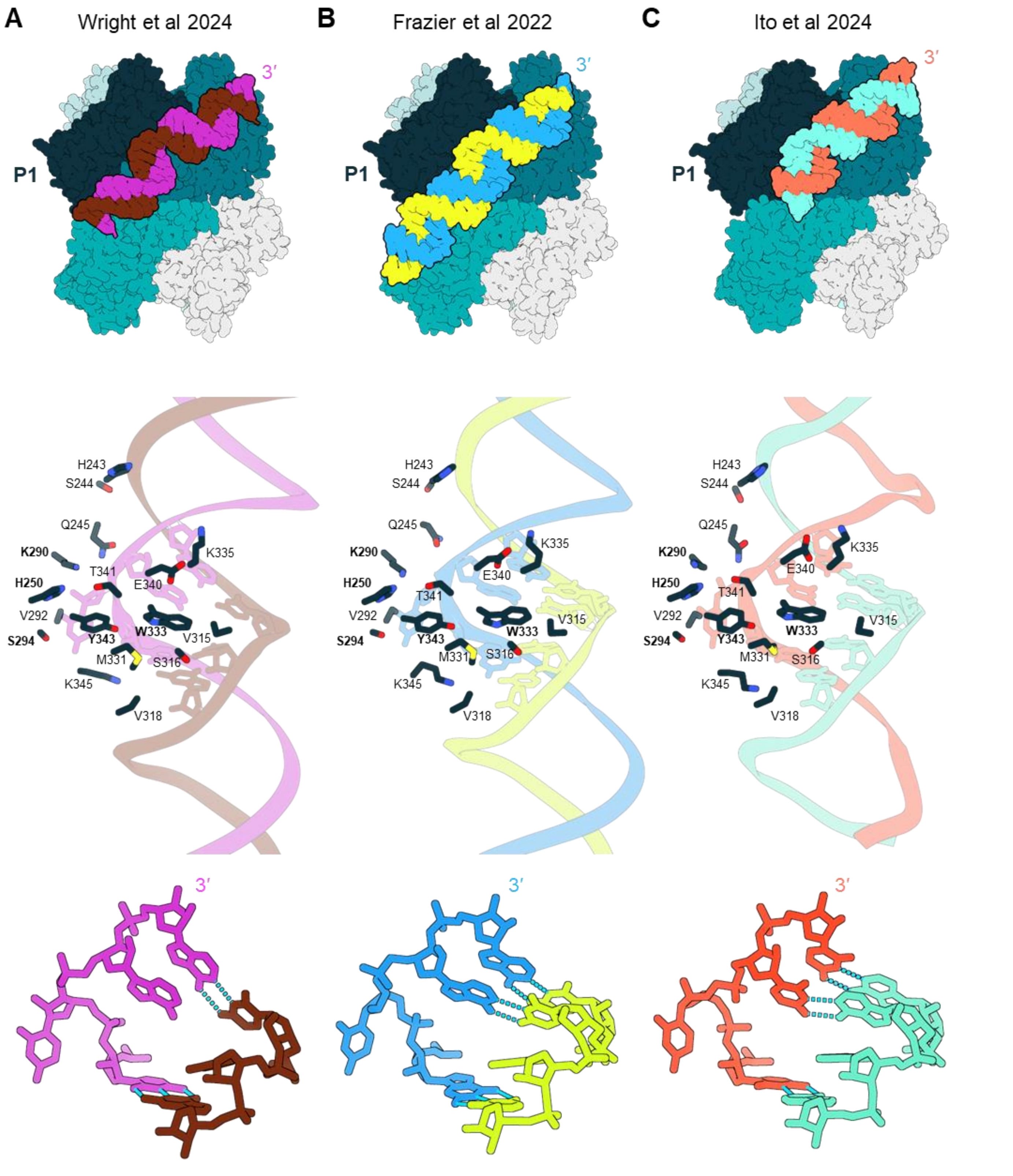

Supplementary Figure 4. Comparison of atomic models by Wright et al (this manuscript A, PDB ID 9BIH), Frazier et al (B, 7TQV)[3], and Ito et al (C, 8UD4)[4], with full model view (top, dsRNA is outlined in black), key amino acid sidechains of Nsp15′s protomer P1 endoU that interact with the dsRNA (middle), and bases immediately neighboring the flipped U that is engaged with Nsp15 (bottom, H-bonds shown as dotted cyan lines). Models were aligned by atoms in protomer P1 of Nsp15 (chain A in all models). Note, residue numbering for Ito et al has been adjusted by +1 here compared to the model file to account for the N-terminal methionine present in models by Wright et al (this manuscript) and Frazier et al.[3]

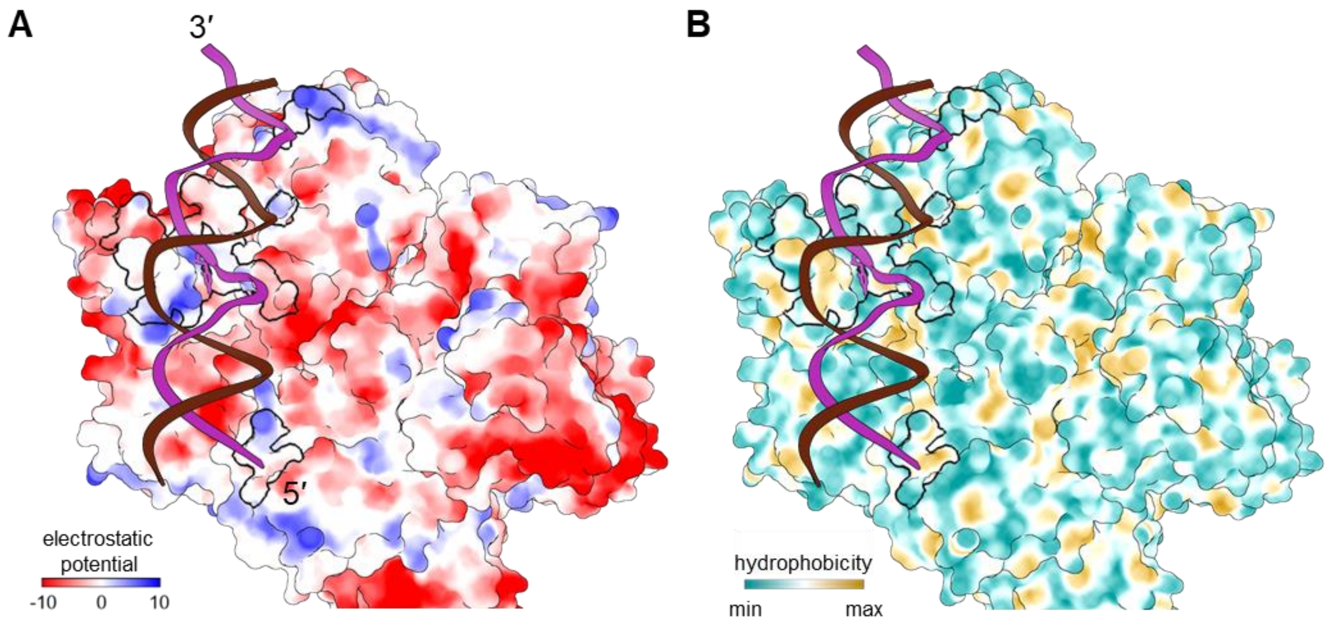

Supplementary Figure 5. Expanded view of A) electrostatic surface potential and B) hydrophobicity (represented by the molecular lipophilicity potential) of Nsp15 around dsRNA binding pocket. As in Figure 3F, residues calculated to interact with dsRNA are outlined in black.

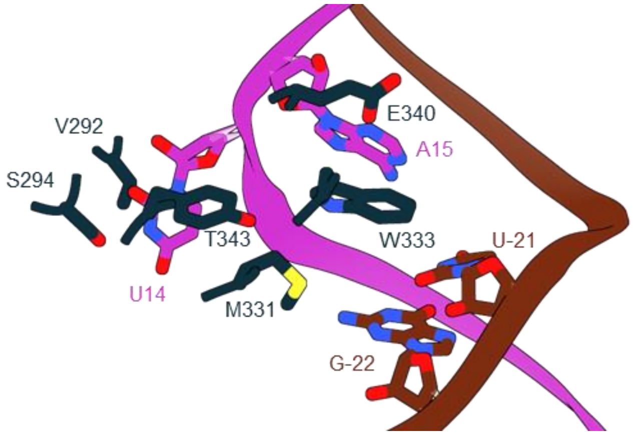

Supplementary Figure 6. Interactions between Nsp15 residues and bases of dsRNA. These interactions are primarily aromatic (W333 with A15 and U-21; T343 with the flipped U14) or hydrophobic (M331 with the sugar edge of G-22; V292 with the flipped U14). S294 distinguishes U from C in the active site of Nsp15. E340 contacts the sugar edge of A15. Interactions with the sugar-phosphate backbone of the dsRNA are omitted as these are not base specific.

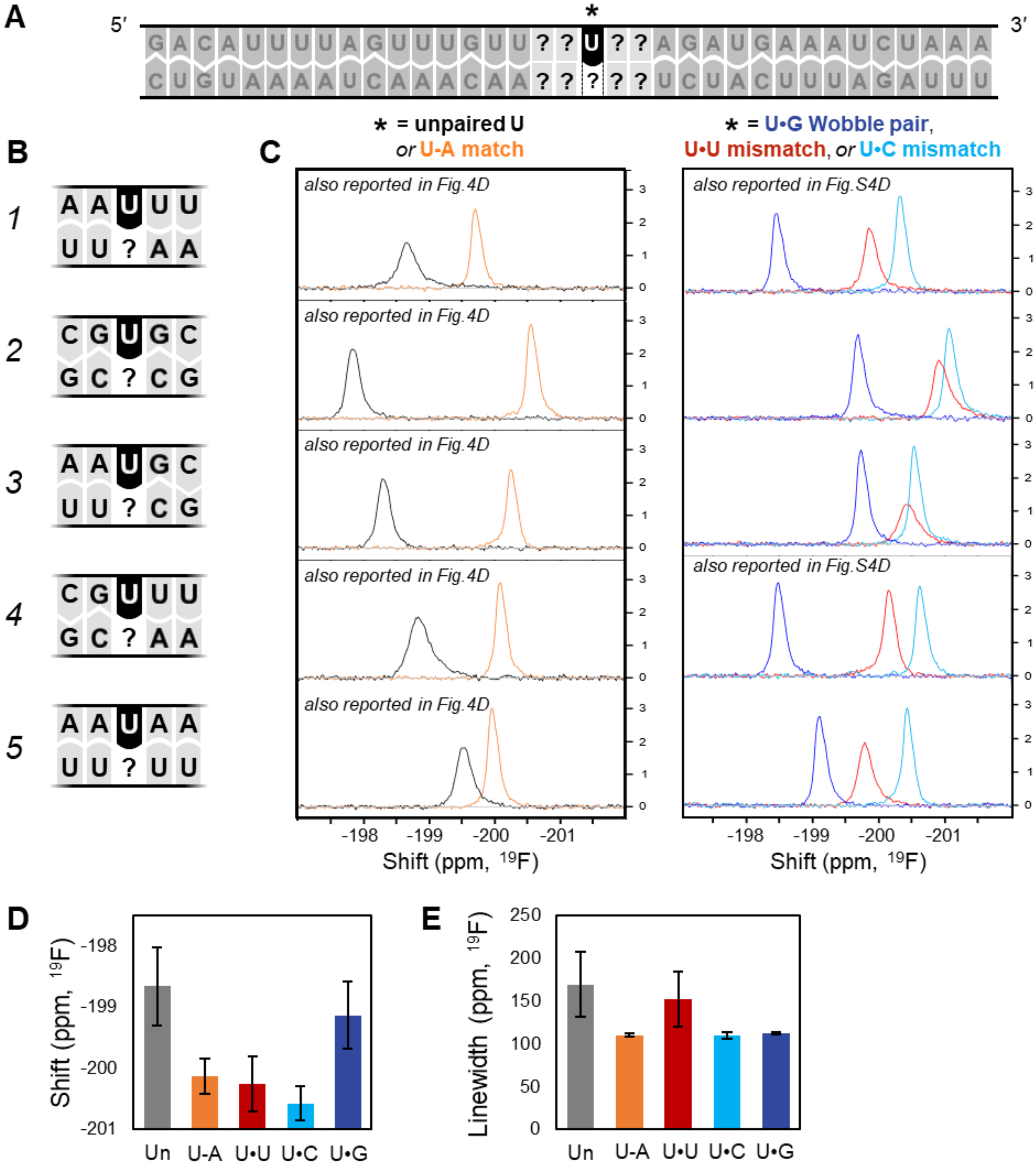

Supplementary Figure 7. Complete set of 1D ^{19}F NMR spectra collected for substrates 1 – 5 and statistics on peak shift and linewidth. A) Substrate design is as described in Figure 4. B) Sequence contexts for observable U*. C) NMR spectra for all sequences and complements tested. Some spectra are reproduced from either Figure 4 or Supplemental Figure 4, as marked, to clarify the complete dataset and to facilitate comparison. D) Average peak shift, in ^{19}F NMR spectra, for each complement. Bar represents average, error bars represent standard deviation of five substrates (i.e., all unpaired substrates with sequences 1 – 5). "Un" stands for unpaired. E) Average linewidth for each complement. Bar represents average, error bars represent standard deviation of five substrates. "Un" stands for unpaired.

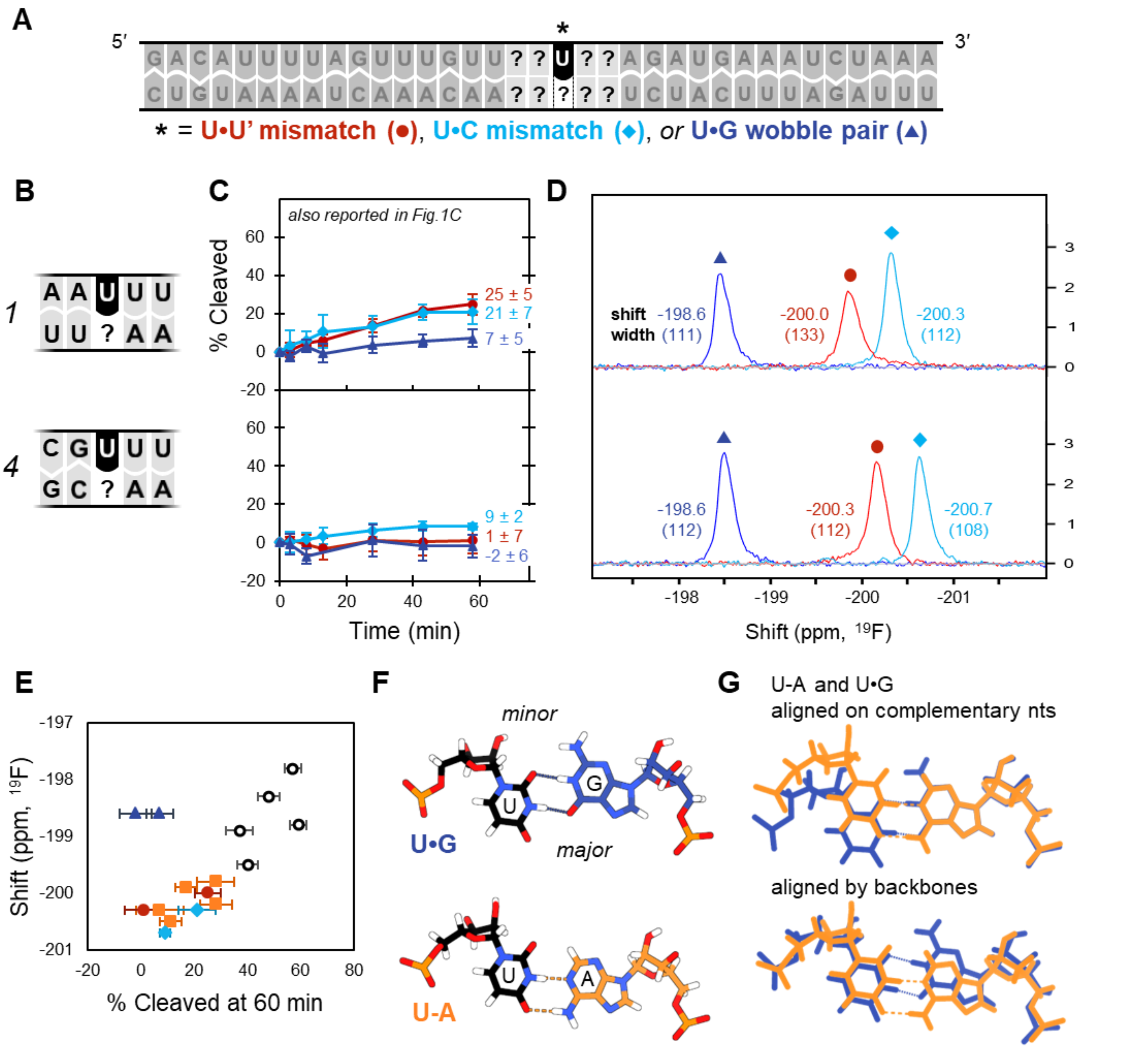

Supplementary Figure 8. A) dsRNA substrate design, with observable target U* (in black). For cleavage assays, U* = 2′-OH, non-target Us in target strand = 2′-F-U. Target strand has 5′ Cy5 and 3′ Fl labels, as in Figure 1. For NMR, U* = 2′-F-U, all other Us = 2′-OH. From this design, a set of substrates were by changing 2 nt on both 5′ and 3′ sides of U* (light gray, marked with ?) to different sequences, shown in B). For each sequence, U* was either engaged in a U•G wobble pair (dark blue), a U•U' mismatch (red) or U•C mismatch (cyan). C) Percent of target strand cleaved over time, quantified via intensity of the uncleaved RNA band and normalized to the 2 min timepoint. Each point with error bars represents average and standard deviation for at least three independent reactions. Data for substrate 1 ("AAUUU") in panel C is reproduced from Figure 1C for clarity and ease of comparison. D) 1D ^{19}F NMR spectra for each substrate. E) Scatter plot of shift (peak position) from 1D ^{19}F NMR spectra vs. % cleaved at 60 min from our enzymatic assays. For each point, x position represents the average of three independent reactions for a single substrate (error bars represent standard deviation), y position represents one spectrum. F) Structural difference between (cis Watson-Watson) U-A and U•G base pairs. G) Superimposed U-A (yellow) and U•G (dark blue) pairs, aligned either by A and G nucleotides (top) or sugar-phosphate backbones (bottom). In U•G pairs, the U is pushed towards the major groove and the G is pushed towards the minor groove, relative to U-A pairs. For panels F and G, the U•G wobble pair is modeled from PDB 8CQ1 (using U87 and G124 from structure #1.5), and the U-A pair is modeled from PDB 7TQV (using U9 from chain g and A44 from chain h).[3,5]

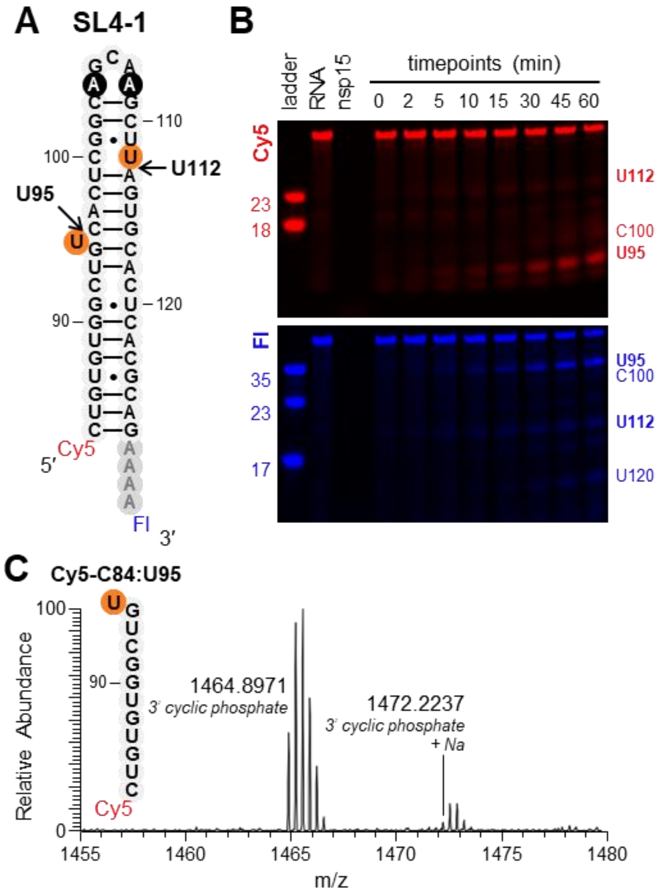

Supplementary Figure 9. Nsp15 cleavage of a mutated stem loop helps confirm the unpaired U in a 1 nt bulge is preferred to other Us. A) Design of substrate with sequence based on the SARS-CoV-2 genome′s Stem Loop 4 (light gray/ orange) with 5′ Cy5 and 3′ A_4-Fl. Nucleotides at positions 104 and 108 (black circles with white text) have been mutated from Us to As. Nts are labeled according to position in the positive strand viral genomic RNA. B) Representative denaturing PAGE gel of time-course nuclease assays, with Cy5 (red) and Fl (blue) channels separated to facilitate band identification. Images of each gel are provided in the Source Data file. C) Mass spectrum showing the multiply charged ions that correspond to Nsp15 digest fragment of the SL4-1 RNA oligonucleotide.

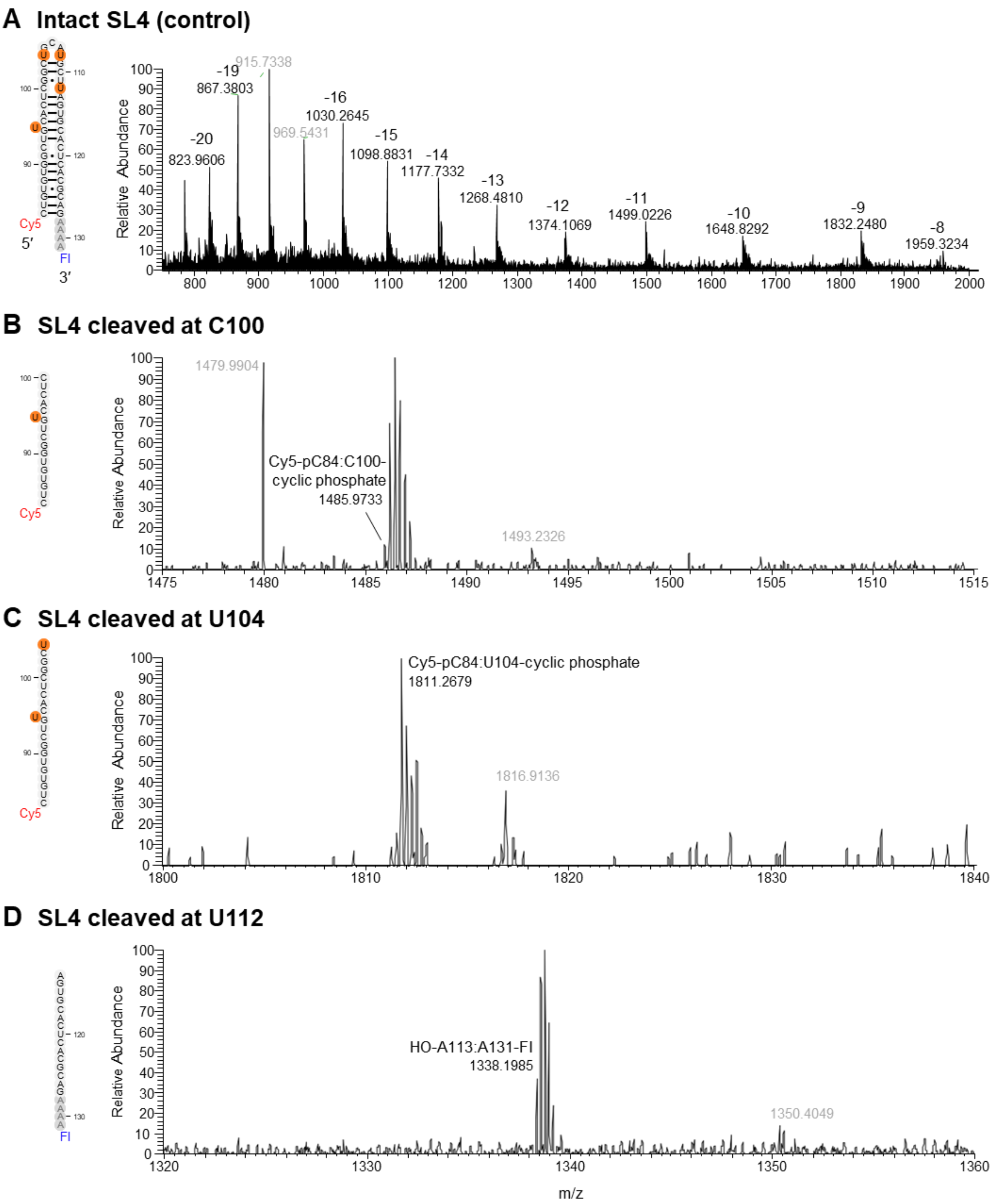

Supplementary Figure 10. Mass spectra showing A) the multiply charged envelope, $(M-8H)^{8-}$ to $(M-21H)^{21-}$, from the analysis of the intact SL4 RNA oligonucleotide; B – D) Mass spectra showing the multiply charged ions that correspond to Nsp15 digest fragments of the SL4 RNA oligonucleotide.

Supplementary References

1 Punjani, A., Rubinstein, J. L., Fleet, D. J. & Brubaker, M. A. cryoSPARC: algorithms for rapid unsupervised cryo-EM structure determination. *Nat Methods* **14**, 290-296 (2017). https://doi.org:10.1038/nmeth.4169

2 Ribeiro, J., Rios-Vera, C., Melo, F. & Schuller, A. Calculation of accurate interatomic contact surface areas for the quantitative analysis of non-bonded molecular interactions. *Bioinformatics* **35**, 3499-3501 (2019). https://doi.org:10.1093/bioinformatics/btz062

3 Frazier, M. N. *et al.* Flipped over U: structural basis for dsRNA cleavage by the SARS-CoV-2 endoribonuclease. *Nucleic Acids Res* **50**, 8290-8301 (2022). https://doi.org:10.1093/nar/gkac589

4 Ito, F., Yang, H., Zhou, Z. H. & Chen, X. S. Structural basis for polyuridine tract recognition by SARS-CoV-2 Nsp15. *Protein Cell* **15**, 547-552 (2024). https://doi.org:10.1093/procel/pwae009

5 Vogele, J. *et al.* High-resolution structure of stem-loop 4 from the 5'-UTR of SARS-CoV-2 solved by solution state NMR. *Nucleic Acids Res* **51**, 11318-11331 (2023). https://doi.org:10.1093/nar/gkad762
