# Supplementary material for: Spontaneous base flipping helps drive Nsp15’s preferences in double stranded RNA substrates
Source: Nat Commun. 2025 Jan 4;16:391. doi: 10.1038/s41467-024-55682-0 (PMC11700208; doi:10.1038/s41467-024-55682-0)
Supplement: Supplementary file 3 — Reporting Summary [file 41467_2024_55682_MOESM3_ESM.pdf]

nature portfolio

Corresponding author(s): Stanley, RE

Last updated by author(s): Nov 26, 2024

Reporting Summary

Nature Portfolio wishes to improve the reproducibility of the work that we publish. This form provides structure for consistency and transparency in reporting. For further information on Nature Portfolio policies, see our Editorial Policies and the Editorial Policy Checklist.

Statistics

For all statistical analyses, confirm that the following items are present in the figure legend, table legend, main text, or Methods section.

n/a	Confirmed	
☐	☒	The exact sample size (*n*) for each experimental group/condition, given as a discrete number and unit of measurement
☐	☒	A statement on whether measurements were taken from distinct samples or whether the same sample was measured repeatedly
☐	☒	The statistical test(s) used AND whether they are one- or two-sided<br>*Only common tests should be described solely by name; describe more complex techniques in the Methods section.*
☒	☐	A description of all covariates tested
☐	☒	A description of any assumptions or corrections, such as tests of normality and adjustment for multiple comparisons
☐	☒	A full description of the statistical parameters including central tendency (e.g. means) or other basic estimates (e.g. regression coefficient) AND variation (e.g. standard deviation) or associated estimates of uncertainty (e.g. confidence intervals)
☐	☒	For null hypothesis testing, the test statistic (e.g. *F*, *t*, *r*) with confidence intervals, effect sizes, degrees of freedom and *P* value noted<br>*Give P values as exact values whenever suitable.*
☒	☐	For Bayesian analysis, information on the choice of priors and Markov chain Monte Carlo settings
☒	☐	For hierarchical and complex designs, identification of the appropriate level for tests and full reporting of outcomes
☐	☒	Estimates of effect sizes (e.g. Cohen's *d*, Pearson's *r*), indicating how they were calculated

Our web collection on statistics for biologists contains articles on many of the points above.

Software and code

Policy information about availability of computer code

Data collection	Polyacrylamide gels were imaged using Amersham Typhoon control software. Cryo-EM data was collected with SerialEM v4.0 or newer.
Data analysis	Cryo-EM data was analyzed with Scipion V3, CryoSPARC v3, and MotionCor2. Model building was carried out in Phenix and Coot. Structure figures were prepared using ChimeraX. Analysis of intermolecular interactions in atomic models was performed in ChimeraX and using the web-based software "dr_sasa," doi: 10.1093/bioinformatics/btz062 (tool accessible online at http://schuellerlab.org/dr_sasa/). NMR data were analyzed with VNMRJ 22.1 (Agilent) and Chenomx 11(Alberta, Canada). Mass spectrometry data were analyzed with the Xcalibur Software Suite (Thermo). RNA gel images were quantified and analyzed with ImageJ and Excel.

For manuscripts utilizing custom algorithms or software that are central to the research but not yet described in published literature, software must be made available to editors and reviewers. We strongly encourage code deposition in a community repository (e.g. GitHub). See the Nature Portfolio guidelines for submitting code & software for further information.

Data

Policy information about availability of data

All manuscripts must include a data availability statement. This statement should provide the following information, where applicable:

- Accession codes, unique identifiers, or web links for publicly available datasets
- A description of any restrictions on data availability
- For clinical datasets or third party data, please ensure that the statement adheres to our policy

The cryo-EM map has been deposited at the EMDB (EMD-44590) and the atomic coordinates have been deposited at the PDB (9BIH). Mass-spec data have been deposited at Massive under the accession code MSV000094614 and can be accessed at the following website: https://massive.ucsd.edu/ProteoSAFe/private-dataset.jsp?task=7cb7509f99544784b373599974a312e0. All other data is provided in the figures or the source data file.

Research involving human participants, their data, or biological material

Policy information about studies with human participants or human data. See also policy information about sex, gender (identity/presentation), and sexual orientation and race, ethnicity and racism.

Reporting on sex and gender	N/A
Reporting on race, ethnicity, or other socially relevant groupings	N/A
Population characteristics	N/A
Recruitment	N/A
Ethics oversight	N/A

Note that full information on the approval of the study protocol must also be provided in the manuscript.

Field-specific reporting

Please select the one below that is the best fit for your research. If you are not sure, read the appropriate sections before making your selection.

☒ Life sciences ☐ Behavioural & social sciences ☐ Ecological, evolutionary & environmental sciences

For a reference copy of the document with all sections, see nature.com/documents/nr-reporting-summary-flat.pdf

Life sciences study design

All studies must disclose on these points even when the disclosure is negative.

Sample size

Cryo-EM:
From one grid, two data collection sessions (one at 0º and one at 30º tilt) yielded a total of 15,494 movies; no area was imaged twice. Particles were picked from all movies with CTF fit resolution < 6 Å (1.8M particles from 13,206 movies). After several classification and refinement steps to separate intact particles with and without dsRNA density, a total of 217,900 particles contributed to the final map, which was sufficient to cover a complete distribution of viewing angles.

Nuclease Assays:
As is standard practice, at least three biological replicates were performed (each with distinct preps of protein) for each condition.

Data exclusions

Cryo-EM:
No particles were picked from exposures with CTF fit resolution calculated to be >6 Å (2,288 exposures rejected of 15,494 collected) as these exposures are not likely to contribute high resolution information to the final map. The cryo-EM sample was expected to contain a mixture of Nsp15+dsRNA, apo Nsp15, free dsRNA, ice, and some fraction of degraded or distorted particles. Established algorithms in CryoSPARC v3 were used to identify potential particles in accepted exposures and sort images of these particles into classes via unbiased (non-templated) procedures. Classes containing visually-identifiable density for Nsp15-bound dsRNA were selected for further refinement, while classes containing ice or missing dsRNA density or large areas of protein were not used further. See methods and Supplemental Figure 1 for more detail.

Nuclease Assays:
When quantifying RNA cleavage from nuclease assay gels, one gel well that was obviously over-filled (intensity much greater than t = 0; both uncleaved RNA and product bands proportionally overly intense) was excluded from data analysis. This affected one timepoint from one replicate in one sample (sample 1G, t = 60 min, both red and blue image channels); four unaffected measurements (two biological replicates each measured in two image color channels) contributed to the final average value reported in the main text. Error bars for this timepoint were estimated conservatively by taking the greatest standard deviation value calculated of all other timepoints of this dsRNA sample (n = 3 biological replicates).

Replication	Cryo-EM:<br>The cryo-EM structure we report in this study is consistent with a previously determined structure of Nsp15-bound dsRNA but with improved resolution in the dsRNA. Though 217,900 particles contributed to our final structure, about twice that many high-quality particles of Nsp15-bound dsRNA were identified during processing. Several iterations of refinements were performed to limit local blurring of the map caused by averaging together structures with slight differences in conformation, but over the course of processing, 3D maps and 2D classes were consistently generated via unbiased (template-free) processes that show Nsp15-bound dsRNA with essentially equivalent structures and relatively small differences in resolution.<br><br>Nuclease Assays:<br>All nuclease assays were performed at least in triplicate with distinct preparations of protein (three biological replicates). To quantify the amount of uncleaved (dual-labeled) RNA over time, band intensity in gels was measured in both red and blue channels; data from both channels were used to calculate % cleavage. Each time an assay was performed, multiple different dsRNA substrates were assayed in parallel against the same prep of protein, to make it possible to distinguish defective or unusual protein preps from real variation within a sample. All replicates were successful with the exception of replicates that were discarded because of minor technical issues with loading and running of gels.
Randomization	Cryo-EM:<br>Once a grid with acceptable particle density and ice thickness was manually identified, data was acquired in automation across many grid squares, providing a random selection of particles. Data processing was performed using standardized algorithms in CryoSPARC v3 that randomly divide particle stacks into seed groups and shuffle the order that particles are analyzed to avoid introducing bias.<br><br>Nuclease Assays:<br>Randomization is not standard for nuclease assays as we are controlling the substrate.
Blinding	Blinding is not standard in either cryo-EM or nuclease assays. To strengthen our assignment of cleavage products for our nuclease assays on substrates with multiple possible cleavage sites (Figure 5, Supplemental Figure 6), assay samples were provided by the first author to our collaborator for mass spectrometry analysis without information about which cleavage products had been observed by PAGE.

Reporting for specific materials, systems and methods

We require information from authors about some types of materials, experimental systems and methods used in many studies. Here, indicate whether each material, system or method listed is relevant to your study. If you are not sure if a list item applies to your research, read the appropriate section before selecting a response.

Materials & experimental systems

n/a	Involved in the study
☒	☐ Antibodies
☒	☐ Eukaryotic cell lines
☒	☐ Palaeontology and archaeology
☒	☐ Animals and other organisms
☒	☐ Clinical data
☒	☐ Dual use research of concern
☒	☐ Plants

Methods

n/a	Involved in the study
☒	☐ ChIP-seq
☒	☐ Flow cytometry
☒	☐ MRI-based neuroimaging

Plants

Seed stocks	*Report on the source of all seed stocks or other plant material used. If applicable, state the seed stock centre and catalogue number. If plant specimens were collected from the field, describe the collection location, date and sampling procedures.*
Novel plant genotypes	*Describe the methods by which all novel plant genotypes were produced. This includes those generated by transgenic approaches, gene editing, chemical/radiation-based mutagenesis and hybridization. For transgenic lines, describe the transformation method, the number of independent lines analyzed and the generation upon which experiments were performed. For gene-edited lines, describe the editor used, the endogenous sequence targeted for editing, the targeting guide RNA sequence (if applicable) and how the editor was applied.*
Authentication	*Describe any authentication procedures for each seed stock used or novel genotype generated. Describe any experiments used to assess the effect of a mutation and, where applicable, how potential secondary effects (e.g. second site T-DNA insertions, mosiacism, off-target gene editing) were examined.*
